# Regulatory Mechanism of LINC00152 on Aggravating Heart Failure through Triggering Fibrosis in an Infarcted Myocardium

**DOI:** 10.1155/2021/2607358

**Published:** 2021-12-01

**Authors:** Lizhong Song, Xiujuan Duan, Xiaojuan Zeng, Xinglian Duan, Li Li

**Affiliations:** ^1^Department of Emergency, Shanxi Cardiovascular Disease Hospital, Taiyuan, Shanxi Province, China; ^2^Department of Cardiology, The Eighth People's Hospital of Hengshui City, Hengshui, China; ^3^Department of Cardiology, The Third Affiliated Hospital of Chongqing Medical University, Chongqing, China

## Abstract

**Objective:**

To elucidate the role of LINC00152 in the progression of heart failure following myocardial infarction. *Patients and Methods*. Serum levels of LINC00152 in acute myocardial infarction (AMI) patients were detected by quantitative real-time polymerase chain reaction (qRT-PCR). Receiver operating characteristic (ROC) curves were depicted for assessing the diagnostic value of LINC00152 in AMI. Subsequently, an *in vivo* AMI model was generated in mice. LINC00152 level in a mouse infarcted myocardium was detected. Echocardiogram was conducted to evaluate the influence of LINC00152 on cardiac function in AMI mice. Primary cardiac fibroblasts were isolated from neonatal mice. After knockdown of LINC00152, proliferative and migratory changes in primary cardiac fibroblasts were assessed by cell counting kit-8 (CCK-8) and transwell assay, respectively. The regulatory effect of LINC00152 on Smad7 level was determined by qRT-PCR. Finally, the involvement of Smad7 in LINC00152-regulated proliferative and migratory abilities in primary cardiac fibroblasts was explored by rescue experiments.

**Results:**

Serum level of LINC00152 was elevated in AMI patients. ROC curves demonstrated the diagnostic potential of LINC00152 in AMI (95% CI: 0.806-0.940, *p* = 0.034). In myocardial tissues collected from AMI mice, LINC00152 level was higher than those collected from mice of the sham group. LVEF and FS markedly decreased in AMI mice overexpressing LINC00152 on the 4^th^ week of AMI modeling. After knockdown of LINC00152 in primary cardiac fibroblasts, proliferative and migratory abilities were declined, which were abolished by Smad7 intervention.

**Conclusions:**

By downregulating Smad7, LINC00152 aggravates heart failure following AMI *via* promoting the proliferative and migratory abilities in cardiac fibroblasts.

## 1. Introduction

Heart failure (HF) is the terminal stage of most cardiovascular diseases. HF severely endangers human health because its incidence and mortality are extremely high. Globally, over 37.7 million people suffer from HF, and its incidence has annually increased. In 2003, there were 4.5 million HF patients in China, including 500,000 new onsets [1]. Acute myocardial infarction (AMI) can result in myocardial necrosis, myocardial fibrosis, and ventricular remodeling, which is an important cause of HF [2]. Although advanced progress has been made on the diagnosis and treatment of HF, the molecular mechanism of HF following AMI has not been clarified. Effective strategies to delay or even cure HF following AMI are urgently required [3].

Long noncoding RNAs (lncRNAs) are linear transcription RNAs with 200 to 100,000 nucleotides long. As a newly discovered type of ncRNAs, lncRNAs are functional to regulate protein expressions, rather than the carries of protein translation [4]. In fact, a large number of lncRNAs are considered the direct or indirect factors influencing gene expressions [5]. Very recently, lncRNAs have been identified as biomarkers and therapeutic targets for AMI [6]. A clinical trial pointed out that expression levels of urothelial tumor-associated lncRNAs in blood circulation are downregulated at 72 h following AMI [7]. Besides, 2810403D21Rik/MirfLncRNA promotes ischemic myocardial injury by regulating autophagy through targeting Mir26a [8]. Further, ZFAS1 as a SERCA2a inhibitor to cause intracellular Ca overload and contractile dysfunction in a mouse model of myocardial infarction [9]. A microarray analysis on AMI mouse tissues showed that two transcriptional lncRNAs associated with myocardial infarction are significantly upregulated by 5 and 13 times, respectively [10].

LINC00152 is located on chromosome 2p11.2, containing 828 nucleotides. It is initially detected in hepatocellular carcinoma with a differentially low methylation level. LINC00152 is able to regulate gene expressions through epigenetic modification [11], lncRNA-miRNA interaction [12], or lncRNA-protein interaction [13]. Serving as an oncogene, LINC00152 is upregulated in many types of malignant tumors [14–16]. The biological function of LINC00152 in AMI and HF is largely unclear. This study is aimed at uncovering the influence of LINC00152 on regulating cardiac fibroblast phenotypes and cardiac function following AMI.

## 2. Patients and Methods

### 2.1. Subjects

A total of 50 eligible AMI patients who were emergently admitted within 6 h of acute chest pain and diagnosed by coronary angiography from May 2017 to December 2019 were enrolled in the AMI group. During the same period, 50 healthy subjects undergoing physical examination were enrolled in the control group. The inclusion criteria of AMI patients were as follows: (1) there are symptoms of chest pain within 6 h; (2) clinical indexes, including ECG findings, myocardial markers, and myocardial enzymes, were in accordance to *2007 ACCF/AHA guideline for the management of ST-elevation myocardial infarction* [17]; (3) coronary angiography showed over 50% of coronary stenosis in more than 1 branch; and (4) this study was approved by the Ethics Committee of The Third Affiliated Hospital of Chongqing Medical University. Signed written informed consents were obtained from all participants before the study. The following are the exclusion criteria: (1) chest pain due to acute trauma, pulmonary embolism, aortic dissection, etc.; (2) complication with liver and kidney dysfunction, heart valve disease, malignant tumors, or others. Venous blood samples were collected in EDTA anticoagulation tubes from each subject and centrifuged at 3,000 r/min for 10 min. The isolated plasma was further centrifuged at 12,000 r/min for 10 min, and the upper layer was collected in RNA-free EP tubes for use.

### 2.2. Generation of AMI Model in Mice

This study was approved by the Animal Ethics Committee of Chongqing Medical University Animal Center. Male C57BL/6 mice (8-10 weeks old) were provided by Charles River (Beijing, China). The AMI model in mice was generated by ligating the anterior descending branch of the coronary artery. Briefly, mice were anesthetized by 1.0%-1.5% isoflurane. Endotracheal intubation connected to the small animal ventilator was conducted. Subsequently, mouse thoracic cavity was exposed from the fourth intercostal space. The parietal pericardium was bluntly separated, and the anterior descending branch of the coronary artery was ligated using 7-0 suture. A pale myocardium in the ligation area indicated the successful modeling. The incision was closed using 5-0 suture. One week prior to AMI modeling, 1.0 × 10^7^ TU LV-LINC00152 (150 *μ*L) or negative control of lentivirus was administrated into mouse tail vein. Transfection efficacy of lentivirus was examined by quantitative real-time polymerase chain reaction (qRT-PCR).

### 2.3. Cardiac Function Assessment

Four weeks after AMI modeling in mice, cardiac function was assessed by performing echocardiography using the Vevo 2100 Small Animal Ultrasound (probe: MS-400). LVEF (left ventricular ejection fraction) and FS (fractional shortening) in mice were recorded.

### 2.4. Cell Culture of Primary Cardiac Fibroblasts and Cell Transfection

The heart of neonatal C57BL/6 mouse within three days after birth was collected and cut into small pieces and digested in the mixture containing 0.08% trypsin and 0.05% collagenase II at 4°C overnight. Digestion was terminated using Dulbecco's modified Eagle medium/F-12 (DMEM/F-12) (HyClone, South Logan, UT, USA) containing 15% fetal bovine serum (FBS) (HyClone, South Logan, UT, USA). The solution was centrifuged at 1000 r for 10 min, and the collected cells were inoculated in the culture dishes for 1 h. Later, adherent cells were primary cardiac fibroblasts.

Primary cardiac fibroblasts were induced in 5 *μ*g/mL Polybrene (YEASEN, Shanghai, China) and transfected with 5 × 10^7^ TU lentivirus for 24 h. Culture medium was replaced, and cells were cultivated for another 48 h. Transfection of si-LINC00152 was conducted using Lipofectamine 2000 (si-LINC00152-1#: 5′-GGGAAATAAATGACTGGAT-3′; si-LINC00152-2#: 5′-GGAGATGAAACAGGAAGCT-3′) (Invitrogen, Carlsbad, CA, USA).

### 2.5. qRT-PCR

Total RNAs were isolated from tissues or blood samples using RNA extraction kit (ABI, Foster City, CA, USA). The concentration and purity of RNA were determined using an ultraviolet spectrophotometer (Thermo Fisher Scientific, Waltham, MA, USA). After reverse transcription, complementary deoxyribose nucleic acids (cDNAs) were amplified for qRT-PCR. Relative mRNA level was calculated by 2^-*ΔΔ*Ct^. Primer sequences were as follows: LINC00152 (human): 5′-TGGGAATGGAGGGAAATAAA-3′ (forward) and 5′-CCAGGAACTGTGCTGTGAAG-3′ (reverse), LINC00152 (mouse): 5′-CAGCACCTCTACCTGTTG-3′ (forward) and 5′-GGATTAAGACACATAGAGACTG-3′ (reverse), and GAPDH: 5′-AACGGATTTGGTCGTATTGG-3′ (forward) and 5′-TTGATTTTGGAGGGATCTCG-3′ (reverse); GAPDH (mouse): 5′-CATCACTGCCACCCAGAAGACTG-3′ (forward) and 5′-ATGCCAGTGAGCTTCCCGTTCAG-3′ (reverse).

### 2.6. Cell Counting Kit-8 (CCK-8)

1.0 × 10^3^ cells were implanted in each well of a 6-well plate and cultured for 1 day, where 10 *μ*L of CCK-8 solution was added (TaKaRa, Dalian, China). After 1 h culturing in the dark, the optical density at 450 nm was measured using a microplate reader.

### 2.7. Transwell Assay

100 *μ*L of serum-free suspension (1.0 × 10^5^ cells/mL) and 600 *μ*L of serum-containing medium were applied to the top and bottom transwell chamber, respectively, and cultured overnight. Cells in the bottom were subjected to methanol fixation for 15 min and crystal violet staining for 20 min. Migratory cells were counted in 5 randomly selected fields per sample.

### 2.8. Statistical Analysis

Data analysis was performed using Statistical Product and Service Solutions (SPSS) 22.0 software (IBM, Armonk, NY, USA). Differences between groups were compared using Student's *t*-test. Receiver operating characteristic (ROC) curves were depicted for assessing the diagnostic potential of LINC00152 in AMI. *p* < 0.05 was considered statistically significant.

## 3. Results

### 3.1. Increased Serum Level of LINC00152 in AMI Patients

**Figure 1:**
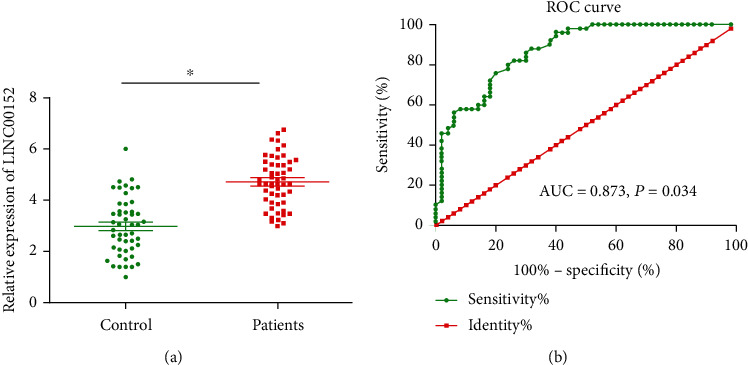
Increased serum level of LINC00152 in AMI patients. (a) Serum level of LINC00152 increased in AMI patients than healthy subjects; (b) ROC curves demonstrated the diagnostic potential of LINC00152 in AMI (AUC = 0.873, *p* = 0.034).

Compared with healthy subjects, the serum level of LINC00152 was markedly higher in AMI patients (Figure 1(a)). Subsequently, the diagnostic potential of LINC00152 in AMI was identified by the depicted ROC curves (AUC = 0.873, 95%CI = 0.806-0.940, *p* = 0.034) (Figure 1(b)). It is suggested that LINC00152 may be a potential biomarker for AMI.

### 3.2. LINC00152 Aggravated HF following AMI

**Figure 2:**
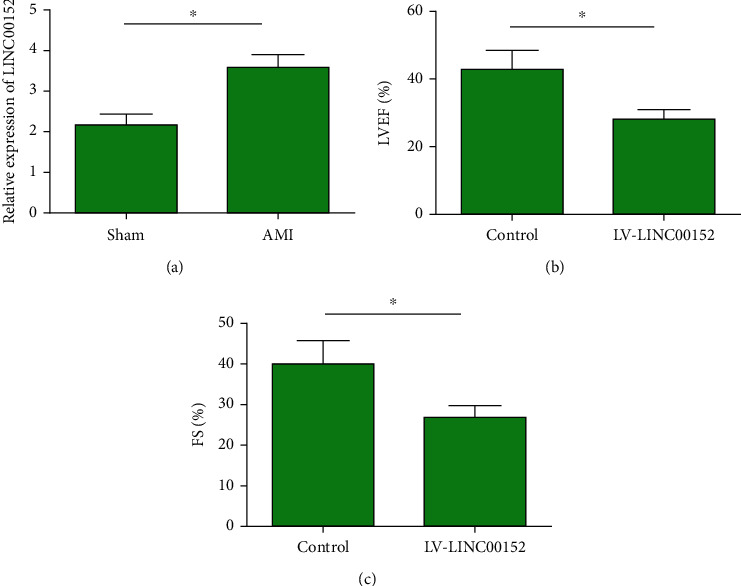
LINC00152 aggravated HF following AMI. (a) LINC00152 was upregulated in mice of AMI group than those in the sham group; (b) LVEF (%) was lower in AMI mice overexpressing LINC00152 than in AMI mice; (c) FS (%) was lower in AMI mice overexpressing LINC00152 than in AMI mice.

To elucidate the influence of LINC00152 on HF following AMI, we generated an *in vivo* AMI model in mice. Myocardial tissues were collected from AMI mice and those in the sham group one week after modeling. Compared those in the sham group, LINC00152 level was much higher in infarcted myocardial tissues collected from AMI mice (Figure 2(a)). Besides, echocardiogram findings uncovered that LVEF and FS were lower in AMI mice overexpressing LINC00152 than those in AMI mice (Figures 2(b) and 2(c)). It is indicated that overexpressed LINC00152 aggravated cardiac function in AMI mice.

### 3.3. LINC00152 Promoted Proliferative and Migratory Abilities in Cardiac Fibroblasts

**Figure 3:**
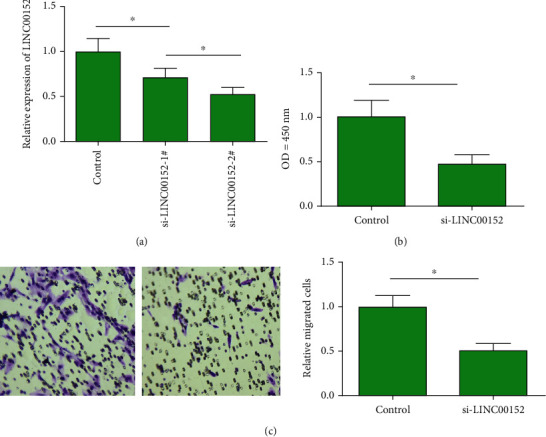
LINC00152 promoted proliferative and migratory abilities in cardiac fibroblasts. (a) Transfection of either si-LINC00152-1# or si-LINC00152-2# significantly downregulated LINC00152 in primary cardiac fibroblasts. (b) Knockdown of LINC00152 weakened proliferation in primary cardiac fibroblasts. (c) Knockdown of LINC00152 weakened migration in primary cardiac fibroblasts (magnification: 200x).

We thereafter explored the *in vitro* function of LINC00152 in the process of AMI. Primary cardiac fibroblasts were used for generating the LINC00152 intervention group. Two lines of LINC00152 siRNAs were constructed. Transfection of either of them could effectively downregulate LINC00152 in primary cardiac fibroblasts (Figure 3(a)). In particular, transfection efficacy of si-LINC00152-2# was better than the other one, which was used in the following experiments. CCK-8 assay uncovered that knockdown of LINC00152 reduced viability in primary cardiac fibroblasts (Figure 3(b)). Besides, transfection of si-LINC00152 in primary cardiac fibroblasts reduced migratory cell number, indicating the attenuated migratory ability (Figure 3(c)).

### 3.4. LINC00152 Regulated Cardiac Fibroblast Functions by Regulating Smad7

**Figure 4:**
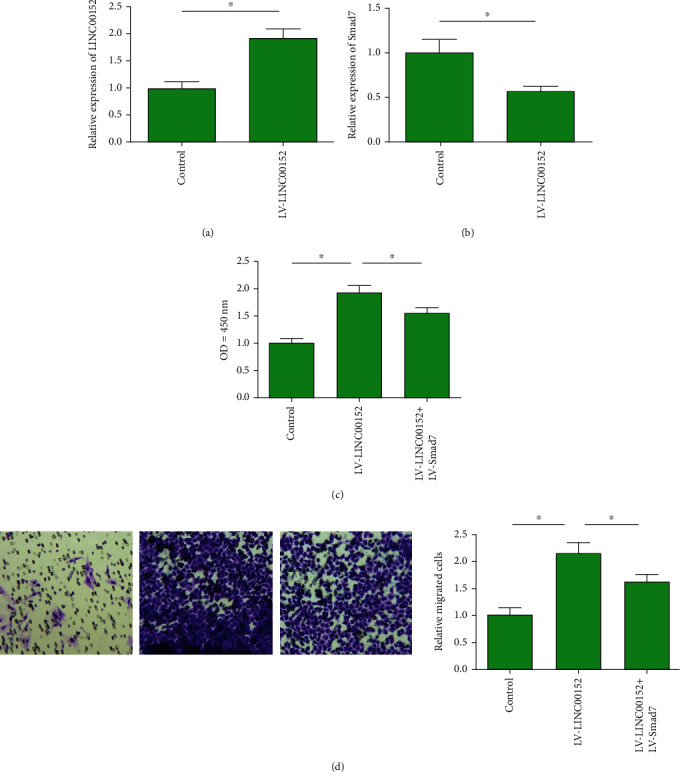
LINC00152 regulated cardiac fibroblast functions by regulating Smad7. (a) Transfection of LV-LINC00152 significantly upregulated LINC00152 in primary cardiac fibroblasts. (b) Overexpression of LINC00152 downregulated Smad7 in primary cardiac fibroblasts. (c) Enhanced proliferation in primary cardiac fibroblasts overexpressing LINC00152 was partially reversed by cooverexpression of Smad7. (d) Enhanced migration in primary cardiac fibroblasts overexpressing LINC00152 was partially reversed by cooverexpression of Smad7 (magnification: 200x).

Transfection of LV-LINC00152 markedly upregulated LINC00152 in primary cardiac fibroblasts, suggesting a great transfection efficacy (Figure 4(a)). In primary cardiac fibroblasts overexpressing LINC00152, Smad7 was downregulated (Figure 4(b)). Interestingly, enhanced viability and migratory cell number in primary cardiac fibroblasts overexpressing LINC00152 were partially reversed by cooverexpression of Smad7 (Figures 4(c) and 4(d)). Collectively, LINC00152 promoted proliferative and migratory abilities in primary cardiac fibroblasts by negatively regulating Smad7.

## 4. Discussion

AMI is the major reason for death in the world [18]. At present, the diagnosis of AMI relies on examinations of previous history, chest pain symptoms, electrocardiogram (ECG) findings, and laboratory tests. Detection of cTNI/cTNT level is extensively applied in the adjuvant diagnosis of AMI. Nevertheless, cTNI/cTNT level is also elevated in non-AMI patients, including viral myocarditis, heart failure, atrial fibrillation, chronic kidney disease, and sepsis [19–21]. Moreover, ECG changes, clinical symptoms of chest pain, and previous history vary a lot in individuals, which are not specific enough for AMI diagnosis. Therefore, it is necessary to seek for serum biomarkers for AMI that can rapidly and stably diagnose AMI in the early phase, thus salvaging the lives.

At post-AMI, cardiac fibroblasts are activated and excessive ECM proteins are released. Damaged myocardial cell functions ultimately lead to interstitial fibrosis and remodeling [22]. Therefore, inhibiting the excessive secretion and deposition of ECM is an important strategy to improve the prognosis of AMI. By detecting differentially expressed lncRNAs in the infarcted myocardium collected from AMI mice at the fourth week, there are 53 upregulated lncRNAs (fold change > 2 times) and 37 downregulated ones. Among them, NONMMUT022554 is the most pronounced lncRNA that is upregulated in the infarcted myocardium, displaying a positive correlation to six upregulated genes that are interacted with ECM receptors [22]. lncRNAs are associated with the development of myocardial fibrosis as well. It is reported that overexpression of H19 triggers proliferation and fibrosis in myocardial fibroblasts [23]. LINC00152 is a cancer-associated lncRNA. In the liver cancer cell line MHCC-97H, LINC00152 is mainly distributed in the nucleus and it drives *in vitro* proliferative rate and *in vivo* cancer growth [24]. Cai et al. [25] demonstrated that LINC00152 stimulates gallbladder cells to proliferate and metastasize, whereas cell apoptosis is inhibited. Wu et al. [26] uncovered that overexpression of LINC00152 induces proliferation and invasiveness in renal cancer cells and inhibits cell cycle progression and apoptosis. In this paper, LINC00152 was upregulated in the serum of AMI patients and the infarcted myocardium of AMI mice. In addition, overexpression of LINC00152 aggravated HF severity following AMI. To clarify the mechanism of LINC00152 on myocardial fibrosis, we isolated primary cardiac fibroblasts from neonatal mice. *In vitro* experiments revealed that overexpression of LINC00152 stimulated proliferative and migratory abilities in primary cardiac fibroblasts.

Smad7 is the classical antagonistic factor for the TGF-*β* signaling, presenting an antifibrosis effect [27]. A previous study reported that lncRNA COL1A2-AS1 alleviates proliferative potential in scar-derived fibroblasts *via* the Smad7 signaling [28]. Here, LINC00152 negatively regulated Smad7 level in primary cardiac fibroblasts. Interestingly, overexpression of Smad7 could partially reverse the role of overexpressed LINC00152 on triggering proliferative and migratory abilities in primary cardiac fibroblasts. In this research, we uncover the role of LINC00152 in AMI via in vivo and in vitro assay; however, the mechanism will be explored more detailed in our next research. Our findings provide experimental references for the clinical treatment of HF following AMI.

## 5. Conclusions

By downregulating Smad7, LINC00152 aggravates heart failure following AMI *via* promoting the proliferative and migratory abilities in cardiac fibroblasts.

## Data Availability

The datasets used and analyzed during the current study are available from the corresponding author on reasonable request.
